# Is palpation essential in the digital era of orthotic designing?

**DOI:** 10.3389/fbioe.2026.1648513

**Published:** 2026-02-19

**Authors:** Komal Chhikara, Scott Morrison, Marie-Luise Wille, Buddhi Herath, Kerrie Evans, Dean Hartley, Müge Belek Fialho Teixeira, Bridget Hughes, Natalie Haskell, Amanda Beatson, Marianella Chamorro-Koc, Judith Paige Little, Sinduja Suresh

**Affiliations:** 1 School of Mechanical, Medical and Process Engineering, Faculty of Engineering, Queensland University of Technology, Brisbane, QLD, Australia; 2 Centre for Biomedical Technologies, Faculty of Engineering, Queensland University of Technology, Brisbane, QLD, Australia; 3 Australian Research Council Training Centre for Multiscale 3D Imaging, Modelling, and Manufacturing, Queensland University of Technology, Brisbane, QLD, Australia; 4 Biomechanics & Spine Research Group at the Centre for Children’s Health Research, Faculty of Engineering, Queensland University of Technology, Brisbane, QLD, Australia; 5 iOrthotics, Brisbane, QLD, Australia; 6 Healthia Limited, Brisbane, QLD, Australia; 7 Faculty of Medicine and Health, School of Health Sciences, The University of Sydney, Sydney, NSW, Australia; 8 School of Architecture and Built Environment, Faculty of Engineering, Queensland University of Technology, Brisbane, QLD, Australia; 9 School of Education, Faculty of Creative Industries, Education, and Social Justice, Queensland University of Technology, Brisbane, QLD, Australia; 10 School of Design, Faculty of Creative Industries, Education and Social Justice, Queensland University of Technology, Brisbane, QLD, Australia; 11 School of Architecture, Industrial Design and Planning, Griffith University, Gold Coast, QLD, Australia; 12 QUT Business School, Centre for Decent Work and Industry (CDWI), Queensland University of Technology, Brisbane, QLD, Australia; 13 QUT Design Lab, Faculty of Creative Industries, Education and Social Justice, Queensland University of Technology, Brisbane, QLD, Australia

**Keywords:** 3D scanning, foot, landmarks, orthotics, palpation

## Abstract

**Introduction:**

Custom foot orthotics require identification of anatomical landmarks in the foot to facilitate accurate alignment and precise foot measurements. Traditionally, these landmarks are identified through manual palpation, however, with the advent of digital scanning, it may not be necessary. Therefore, this study aimed to determine whether guidance from manual palpation to identify anatomical landmarks affects (a) reliability of foot measurements and (b) consistency in digitally designed orthotic insoles.

**Methods:**

For reliability, 3D foot scans were obtained under non-weight-bearing (NWB) (n = 24) and weight-bearing (WB) (n = 24) conditions from 12 healthy adult participants (9 females and 3 males; age 29 ± 4 years; height 164 ± 8 cm; weight 60 ± 6 kg). 15 key dorsal and plantar foot measurements were extracted based on the palpation-guided and scan-derived landmarks. Following this, a preliminary assessment of orthotic design consistency was evaluated using a reference participant (n = 1), with 24 orthotic insoles designed (3 designers designed insoles using scan-derived and palpation-guided landmarks (by 3 podiatrists), in NWB and WB).

**Results:**

Intra- and inter-user reliability of palpation-guided landmarks were good to excellent (ICC = 0.90–1). Measurements for scan-derived landmarking showed good to excellent intra-user reliability (0.83–0.92) but poor to moderate inter-user reliability (0.31–0.75) for clinically relevant plantar measurements.

**Discussion:**

While palpation improves landmark reliability, translating these into consistent orthotic designs requires clinical expertise and standardised design workflows. Palpation, while not always essential, improves landmarking identification in variable clinical conditions. Collaborative training and further studies across varied clinician and designer experience levels are essential to optimize digital orthotic design.

## Introduction

Foot orthotics plays an integral part in the management of various musculoskeletal conditions affecting the lower extremities (Choo et al., 2020; Collins et al., 2007). Foot orthotics, or orthotic insoles, typically aim to redistribute plantar pressure and support the arch of the foot. Orthotic insoles can be fabricated either traditionally using Plaster of Paris (PoP) or digitally using three-dimensional (3D) scanning and additive manufacturing (Rogati et al., 2021; Powers et al., 2022; Lee et al., 2014; Farhan et al., 2021; Barrios-Muriel et al., 2020). The fabrication process begins with careful examination and assessment of the patient, their lower limb mechanics, and foot condition to ensure the capture of accurate geometric information for a well-fitted orthotic insole (Landorf and Keenan, 2000). This assessment includes marking key anatomical landmarks in the foot to measure the foot geometry for guiding the orthotic fabrication process (Telfer et al., 2012).

Landmark identification is central to both traditional and digital fabrication methods. In traditional fabrication, the anatomical landmarks are identified by experienced clinicians through manual palpation to help measure the foot using callipers and measuring tape (Mohammed and Albadwi, 2024; Telfer and Woodburn, 2010). Whereas the digital manufacturing approach streamlines the traditional process by using 3D surface scanning as a reliable, efficient, and precise method to capture foot and ankle morphology (Rogati et al., 2021; Powers et al., 2022; Lee et al., 2014; Farhan et al., 2021; Barrios-Muriel et al., 2020). This technology replicates the foot data as 3D volumes/meshes which are used to design customized orthotic insoles. 3D meshes of the foot can be measured by orthotic designers easily in a digital environment using digital landmarks. The selection of these landmarks is based on their distinctiveness, consistency across different foot models, and the algorithms of the orthotic design software program. Few anatomical landmarks, such as navicular tuberosity, and medial and lateral malleoli, can be identified more easily than the others such as the head of metatarsals and the centre of the heel, which may require training to locate correctly on a digital scan. Collectively, these landmarks are used to guide decision-making during the fabrication process (Lee et al., 2014; Kouchi and Mochimaru, 2011; Kouchi et al., 1999). The key digital landmarks needed to design an orthotic insole include the head of the first and fifth metatarsal and centre of the heel on the plantar region and the highest point of the medial longitudinal arch (Telfer et al., 2012; Telfer and Woodburn, 2010).

Anatomical landmarks in the foot are used as fixed reference points for registration to assist accurate alignment for generating a measurement axis, to enhance the reliability and accuracy of foot measurements (Telfer and Woodburn, 2010; Wu et al., 2018; Witana et al., 2006; Barisch-Fritz et al., 2014; Goonetilleke et al., 2009). Once the model is registered, the user calculates distances and circumferences with the help of these predefined landmarks. These measurements form the foundation for modifying the custom orthotic insole tailored to the specific needs of individuals (Lee et al., 2014; Farhan et al., 2021; Telfer and Woodburn, 2010). Landmark identification in 3D models can follow one of three primary approaches:

- Palpation-guided approach- The anatomical landmarks can be marked manually through palpation by clinicians before the 3D scanning process for later identification in the digital designing process. These visible landmarks can then be selected in the 3D scans for accurate alignment with the world coordinate system (Wu et al., 2018; Witana et al., 2006; Xiong et al., 2009; Luo et al., 2009).
- Scan-derived approach- This can be implemented in two ways: by manually placing landmarks on the bony anatomical landmarks in the digital scan (Pantazi and Vasilescu, 2025; Van den Herrewegen et al., 2014) or through automatic detection of bony landmarks using software tools (Rogati et al., 2021; Wu et al., 2018; Wang et al., 2018). The manual method relies on the user’s discretion to identify and place landmarks directly within the 3D model, requiring sufficient training and anatomical knowledge. In contrast, the automated approach detects landmarks without manual input, using techniques such as geometric analysis, statistical shape models, or deep learning algorithms.

Studies have demonstrated that changes in foot morphology can be measured using landmarks, whether palpated (Wu et al., 2018; Witana et al., 2006; Xiong et al., 2009; Luo et al., 2009), manually scan-derived (Pantazi and Vasilescu, 2025; Van den Herrewegen et al., 2014) or Artificial intelligence (AI)-based (Rogati et al., 2021; Wu et al., 2018; Wang et al., 2018). Recent advances in commercial 3D scanning systems, including tablet-based scanners, increasingly incorporate basic automated or AI assisted landmarking algorithms. While AI-based approaches are promising for their potential to automate landmark identification, they require extensive validation to ensure their reliability and clinical applicability, especially in the foot orthotic industry, where the foot morphology could be affected by the pathology (Yahara et al., 2005). Moreover, many of the current AI and computational techniques rely on advanced imaging modalities such as radiographs (Shelton et al., 2019; Noh et al., 2024; Ryu et al., 2022) or CT scans (Subburaj et al., 2009), and require complex software capable of geometric analysis (Rogati et al., 2021), statistical shape modelling (Schuster et al., 2021), or deep learning algorithms (Ryu et al., 2022). These approaches limit their feasibility for routine use in podiatry clinics and orthotic manufacturing settings where time, budget, and technical expertise are limited. Consequently, despite increasing adoption of automated tools, manual landmark identification, either through palpation or clinician-guided, remains the current gold standard, particularly in complex or pathological cases. Therefore, there remains a gap between research advancements and their translation into clinical and commercial orthotic practice (Choo and Chang, 2023; Williams et al., 2016). Consequently, this study will disregard the automated landmark detection technique and consider the manual identification method as the scan-derived approach.

The use of palpation-guided and scan-derived anatomical landmarks is common in both research and clinical settings. Studies have testified to the use and importance of anatomical landmarks to generate accurate measurements (Lee et al., 2014; Witana et al., 2006; Barisch-Fritz et al., 2014; Goonetilleke et al., 2009; Zhao et al., 2008; Liu et al., 1999; Bunch, 1988; Tsung et al., 2003). Clarkson et al. (Clarkson et al., 2016) reported in their study that the palpation-guided identification process is time-consuming but most reliable and yields the greatest accuracy. Similar results were observed by Lee et al. (2014) in their study, reporting that the palpation-guided approach performed better when compared to other traditional methods (digital callipers, and digital and ink footprint) of generating foot measurements. A review study conducted by Telfer and Woodburn (2010) summarized the use of 3D scanning technologies in custom orthotic development and reported that bony landmarks identified on the foot using markers that can be seen in the 3D scans appear to be the most reliable method to obtain girth measurements. However, this accuracy is not without limitations. Errors may arise from both the practitioner’s expertise in locating landmarks manually and the designer’s accuracy in selecting those points digitally, potentially compounding the overall measurement error (Lee et al., 2014; Kouchi and Mochimaru, 2011; Kouchi et al., 1999; Clarkson et al., 2016).

While scan-derived methods are time-efficient and support remote assessments, their accuracy remains uncertain in comparison to manual-palpated methods for orthotic designing. Consequently, the implication of using palpation-guided or scan-derived landmarks in orthotic design remains unclear. It is crucial to address this as orthotic devices are most effective when customized precisely to an individual’s anatomy (Telfer et al., 2012). Moreover, understanding the impact of different weight-bearing positions on anatomical landmark identification is crucial, as foot scans in clinical practice are often captured in varying weight-bearing conditions depending on the individual’s needs or clinician’s preference such as non-weight-bearing (NWB), partial weight-bearing (PWB), half weight bearing (HWB), full-weight bearing (FWB), each reflecting different levels of load on the foot. These positional differences can influence the visibility and accuracy of landmark placement, whether palpation-guided or scan-derived, which may in turn affect foot measurements and the precision of orthotic designs (Chhikara et al., 2025).

With the growing integration of digital technologies in orthotic practice, podiatrists are now starting to perform 3D foot scans and transmit the digital models to orthotic fabrication laboratories. Equipped with additive manufacturing technologies, these facilities rely on in-house orthotic designers to develop customized orthotic insoles based on the scanned data and clinical specifications provided by the podiatrist. Although this division of responsibilities enhances workflow efficiency, it may potentially introduce challenges in translating clinical information between podiatrists and designers. Accuracy in identifying anatomical landmarks may vary between podiatrists (possessing critical anatomical and biomechanical knowledge) and designers (possessing expertise in 3D modelling and digital design software). This disparity in expertise highlights the need to critically examine how effectively anatomical information is communicated from clinics to fabrication laboratories.

While previous studies have examined scanner accuracy, foot measurement reliability, or weight-bearing effects independently, none have evaluated how landmarking method (palpation-guided, scan-derived), scan condition (NWB, WB), and user background (clinicians, designers) collectively influence the reliability of anatomical measurements and the downstream consistency of orthotic designs. Although the analysis of foot morphology is common in the literature, the corresponding orthotic designs are rarely examined. This study provides the first integrated assessment of variability across the digital orthotic workflow, from landmark placement to orthotic designing, thereby identifying how discrepancies propagate into final device geometry. By quantifying the sources of variability, the study contributes new evidence to support the development of standardised digital orthotic design protocols that minimise user-dependent differences and improve design consistency.

## Methodology

This study was conducted in two phases. The primary study focused on reliability of digital foot measurement and the secondary preliminary study focussed on analysing the consistency of digital foot orthotic designs.

### Reliability of foot measurements

#### Study participants

12 healthy participants between the ages of 18–45 years, with no history of using orthotic devices were recruited and both their feet were scanned in two weight bearing conditions. The mean age of the participants was 29 ± 4 years, with mean weight of 60 ± 6 kg and mean height of 164 ± 8 cm (all values reported as mean ± standard deviation). The participants were scanned by the same person throughout the study for consistency.

#### Ethics statement

This study was conducted in accordance with the Declaration of Helsinki and the approval to conduct the study was granted under the title ‘Volumetric scanning of the foot for design of orthoses’ by the Queensland University of Technology Human Research Ethics Committee (# 6097). Informed consent was obtained from all the participants before recruitment. The participants also provided consent for the inclusion of their images in publishing.

#### Testing users

Three users were recruited to test reliability of foot measurements; each had a distinct skill set relevant to the study. The difference in skill set was chosen to analyse the impact of anatomical knowledge and 3D design expertise in landmark identification. The first user (A) had limited experience in 3D modelling (1 year) but possessed extensive knowledge of foot anatomy as an orthotist (6 years). The second user (B) had an expert level of proficiency in 3D modelling (5 years) and had some knowledge of foot anatomy (2 years). The third user (C) also had expert proficiency in 3D modelling (5 years) but had no prior knowledge of foot anatomy. In this study, user C was provided with basic instruction on identifying the MT1 and MT5 and the heel prior to landmark placement. The knowledge of 3D modelling is crucial for testing emerging technologies and their applicability in the orthotic designing field. This range of expertise was selected to reflect the decision-making of users with different experience and knowledge, including early-career or users with limited anatomical training.

#### Study design

**FIGURE 1:**
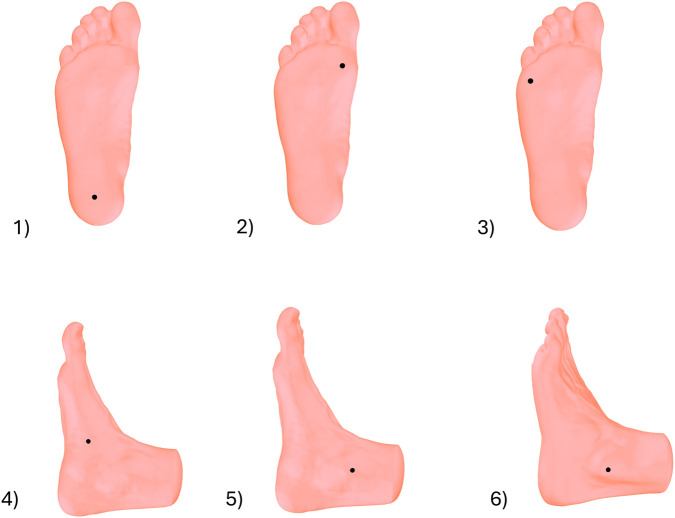
Anatomical landmarks placed on foot before scanning; (1) the centre of the heel, (2) the head of the first metatarsal (MT1), (3) the head of the fifth metatarsal (MT5), (4) the highest point on the medial longitudinal arch, (5) the medial malleolus and (6) the lateral malleolus.

To evaluate reliability of foot measurements, six anatomical landmarks were identified through manual palpation and were marked with 3D stickers. These landmarks included (1) the centre of the heel, (2) the head of the first metatarsal (MT1), (3) the head of the fifth metatarsal (MT5), (4) the highest point on the medial longitudinal arch, (5) the medial malleolus and (6) the lateral malleolus as illustrated in Figure 1.

**FIGURE 2:**
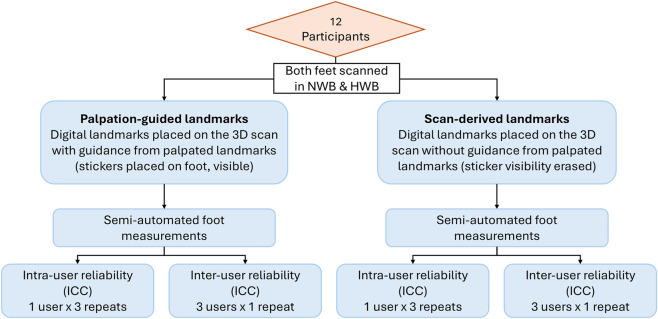
Flowchart of methodology to analyse reliability of foot measurements.

These landmarks were visible and identifiable in the 3D scans and were used to generate fifteen measurements of the foot that were most frequently reported in the literature (Rogati et al., 2021; Allan et al., 2023; Danckaers et al., 2024). The malleoli landmarks were used to assess the effect of different weight bearing on measurements pertaining to the talocrural joint. The weight bearing positions used in this analysis were NWB and HWB. These positions were chosen to capture the likely maximum range of changes in foot measurements between two contrasting weight bearing conditions. Figure 2 outlines the methodology for this study.

#### Scanning process

**FIGURE 3:**
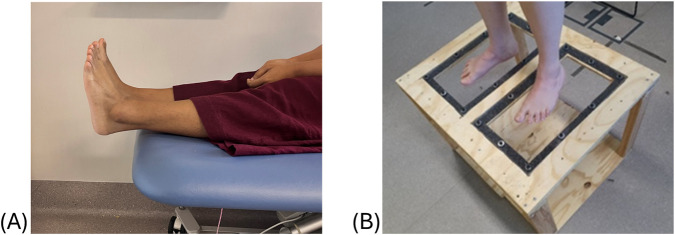
Scanning positions for reliability analysis of foot measurements, **(A)** NWB and **(B)** HWB.

The scanning process for testing the reliability of foot measurements was performed using a structured white light scanner Artec Leo (Artec Group, Senningerberg, Luxembourg), a reliable and accurate method of capturing anthropometric data (Seifert and Griffin, 2020; Modabbe et al., 2016; Juhnke et al., 2019), on both feet of all participants. For the NWB position, participants were asked to sit on a plinth with their feet extended beyond the edge for approximately 1 minute while the scan was completed. An orthotist ensured the foot was positioned correctly and maintained the ankle in a neutral position, while participants were instructed to hold this position throughout the scan, which was completed in under 30 s. For the HWB position, participants were asked to stand still with arms relaxed on their side and body upright by placing their feet on the Perspex glass embedded in the top surface of a transparent podium for a period of one to 2 minutes while the 3D scan of their feet was captured. They were instructed by the orthotist to distribute their weight equally across both legs and maintain a natural stance. To avoid any interference from the Perspex glass, the scans for HWB position were taken in two-halves: a dorsal half from above the platform and a plantar half taken from below the platform. The two-halves were then stitched in the Artec 17 Professional software and saved as a watertight mesh in. STL format files. The scanning positions are illustrated in Figure 3.

**FIGURE 4:**
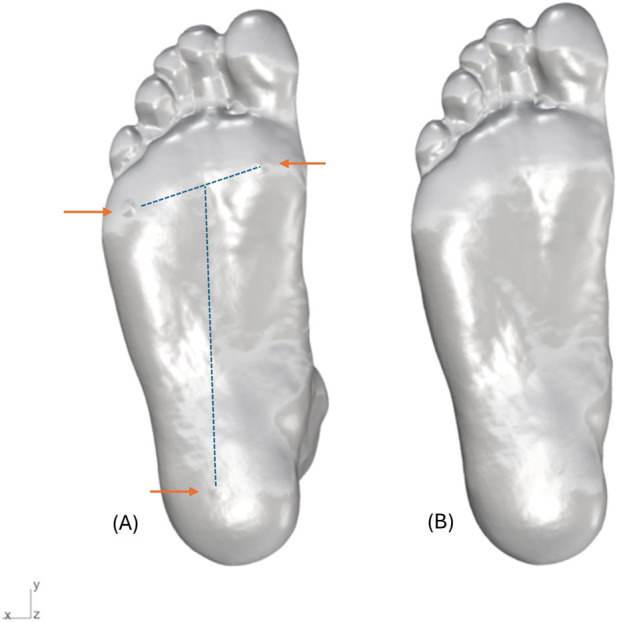
**(A)** Foot scan with palpated landmarks visible (indicated with arrows) and **(B)** Foot scan without palpated landmarks. The local foot coordinate system is shown with origin at the centre of heel; X-axis mediolateral (MT1 to MT5), Y-axis longitudinal (heel to centre of MT1 and MT5), Z-axis vertical (dorsal-plantar).

For the palpation-guided approach, the scans obtained with visibility of 3D stickers were analysed. To analyse the scans for the scan-derived landmark approach, the visibility of 3D stickers was digitally removed from the foot scans using a combination of defeature tool in GeoMagic (version 2023.1.0, Oqton, United States of America) and robust smooth tool in Meshmixer (Autodesk, Inc., San Rafael, CA, United States of America). This step involved minimal processing to maintain the maximum mesh information (Figure 4).

#### Algorithm development

A semi-automated measurement tool that allowed for parametric user input was developed on the 3D modelling and graphical programming software Rhinoceros 3D (Version 7) with Grasshopper add-on (Robert McNeel and associates, United States of America). The 3D mesh model can be manipulated on Rhino while the user inputs to the algorithm are accepted through the Grasshopper interface.

**FIGURE 5:**
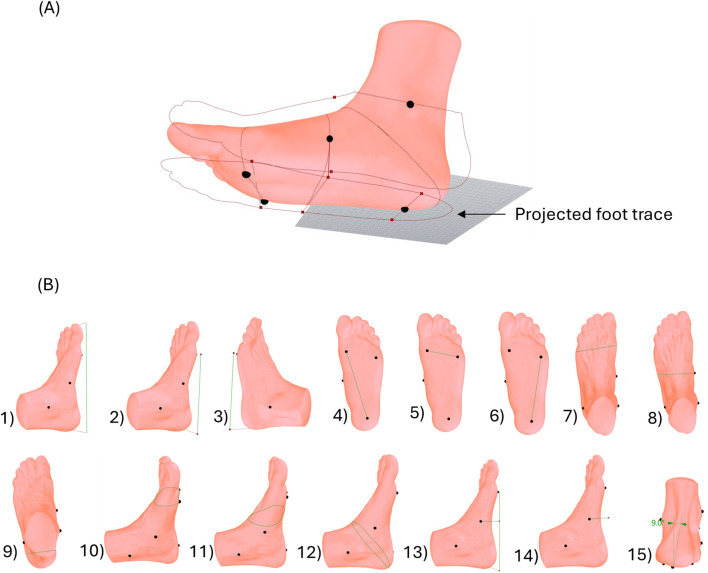
**(A)** Measurement calculation and projected foot trace in Rhino Grasshopper (landmarks displayed as black dots), **(B)** Foot measurements indicated by the green lines; 1) Foot length, 2) Length from base of heel to MT1, 3) Length from base of heel to MT5, 4) Distance between MT1 to heel, 5) Distance between MT1 to MT5, 6) Distance between MT5 to heel, 7) Forefoot width, 8) Midfoot width, 9) Heel width, 10) Forefoot girth, 11) Midfoot girth, 12) Hindfoot girth 13) Arch height index, 14) Arch height, and 15) Ankle angle.

The entire measurement calculation process took approximately 30 s to complete. After importing the foot model as. STL file into the Rhino workspace, the user was required to assign points on the six key anatomical landmarks guided by the 3D sticker markers visible on the scan. For the foot scans with no visible landmarks, the user was required to place points with their own discretion. The three landmarks on the plantar region of the foot were used to align the foot with the base plane. The algorithm first creates a plane using the three user-placed plantar landmarks. The heel landmark was designated as the origin, the longitudinal axis line passing from the heel landmark to the centre of MT1 and MT5 landmarks (midpoint of the line joining the two points) was assigned as the Y-axis, and the Z-axis was oriented in the direction of the ankle. This local reference plane was then used to orient the model to the world coordinate system by matching the origin at the heel and the origin of the World XY plane, the axis line of the foot to the Y-axis of the World XY plane and the normal vector of the world XY plane was used to orient the model in the positive Z-axis direction. The developed algorithm then automatically generated 15 measurements in the foot from these points as displayed in Figure 5. These measurements were then exported to MS excel for further analysis.

The method used to calculate each measurement is detailed in Table 1 below:

**TABLE 1:** Methodology to calculate measurements in the Rhino Grasshopper software.

S.No.	Name of measurement	Method for calculation
1	Foot length	A bounding box was created around the aligned foot scan, trimming the foot scan from the malleoli points. The length of box was extracted as the length of the foot
2	Length from base of heel to MT1	The point on MT1 was projected on the medial aspect of the bounding box. The distance between the base of the bounding box and the projected MT1 point was extracted as the length from base of heel to MT1
3	Length from base of heel to MT5	The point on MT5 was projected on the lateral aspect of the bounding box. The distance between the base of the bounding box and the projected MT5 point was extracted as the length from base of heel to MT5
4	Distance between MT1 to heel	The distance between the marked heel and MT1 points
5	Distance between MT1 to MT5	The distance between the marked MT1 and MT5 points
6	Distance between MT5 to heel	The distance between the marked MT5 and heel points
7	Forefoot width	Landmarks on MT1 and MT5 were projected onto the foot trace and connected by a line. The length of this line was used to represent the forefoot width, corresponding to the width of the metatarsal heads in the projected trace
8	Midfoot width	The arch point was projected onto the foot trace in both medial and lateral directions, and the width of the foot at this location was measured to represent the midfoot width
9	Heel width	The heel point was projected onto the foot trace in both medial and lateral directions, and the width of the foot at this location was measured to represent heel width
10	Forefoot girth	The girth around the metatarsal heads was calculated using the MT1 and MT5 points and was extracted as ball circumference
11	Midfoot girth	The girth of the foot scan was calculated around the midpoint of heel and MT1 to be extracted as midfoot circumference
12	Hindfoot girth	A midpoint was calculated between medial malleoli and arch point. A diagonal circumference was calculated around the ankle with an inclined plane passing through the end of heel and the created midpoint
13	Arch height index	This measurement was calculated as ratio of the arch height to the truncated foot length (length from base of heel to MT1). Literature reports various methods to calculate the arch height index measurement (Zhao et al., 2008; Liu et al., 1999; Bunch, 1988; Tsung et al., 2003; Clarkson et al., 2016; Chhikara et al., 2025; Allan et al., 2023), hence, to keep this calculation consistent, the points assigned by the user were used to calculate the arch height index ratio
14	Arch height	The distance between the base plane to the marked arch point
15	Ankle angle	The heel point was projected in the upward direction. A midpoint was calculated between the medial and lateral malleoli points. The ankle angle was determined as the angular deviation between the line connecting these points and a defined reference line as ankle angle

An overview of the developed algorithm is provided in Supplementary Figure S1 showing the user interface.

#### Reliability analysis

To assess reliability of foot measurements, the three testing users (A,B,C) generated these measurements, thus allowing for analysis of the influence of 3D modelling skill level and knowledge of foot anatomy. The 24 foot scans (both feet of 12 participants) each scanned in two weight bearing positions were then used to evaluate the reliability of intra-user and inter-user. Reliability of foot measurements was evaluated for palpation-guided and scan-derived landmarks.

For the palpation-guided landmark approach, to assess the intra-user reliability, a single user (A) performed three independent repetitions of the measurement process on the same set of foot scans, using the marked anatomical landmarks as reference points. Following this, to assess the inter-user reliability, three different users (A,B,C) independently measured the same foot scans, each conducting a single attempt. This analysis aimed to determine the degree of consistency between same and different users when using the same guided landmarks as reference for measurements.

For the scan-derived landmark approach, the users (A, B, C) were asked to estimate the location of anatomical landmarks in the defeatured foot scans for this phase. The measurements were then repeated under the same conditions, intra-user and inter-user. This process enabled to analyse the comparison between measurements taken with and without manually palpated anatomical landmarks.

### Consistency of orthotic designs

#### Study participants

One healthy participant from the above cohort was recruited as a case study to conduct a preliminary investigation of consistency in orthotic designs. The participant was re-scanned in two weight bearing conditions to evaluate the influence of landmarking methods on orthotic design outcome. By focusing on a single participant, the study aimed to eliminate inter-participant anatomical variability and isolate the specific effects of landmarking methods on consistency of resulting orthotic designs.

#### Testing users

Three trained podiatrists, all familiar with industrial orthotic insole design were recruited to manually place palpated anatomical landmarks on the participant’s foot. Three trained orthotic designers, all with expert knowledge of the orthotic insole design software were recruited for the digital orthotic insole designing process.

#### Study design

A total of 24 orthotic insoles were designed. The participant was scanned in two weight bearing conditions: NWB and PWB. These weight bearing conditions are frequently used to scan patients when designing orthotic insoles in clinical settings. Although HWB and PWB represent different loading protocols with distinct mechanical characteristics, they were selected based on their relevance to the specific objectives of each study phase rather than to represent mechanically equivalent conditions. Phase 1 focuses on measurement reliability under a bilateral standing HWB condition, reflecting common clinical assessment practice. In contrast, Phase 2 examines orthotic design consistency using a controlled PWB setup that is routinely employed in clinical foot-scanning workflows. The first set of foot scans were captured without any manually palpated landmarks in NWB and PWB positions. The foot was then marked with stickers on four anatomical landmarks after manual palpation from three trained podiatrist in succession: MT1, MT5, centre of heel and highest point on medial longitudinal arch. These four landmarks were the requisite for designing orthotic insole in the orthotic design software used in this study. The landmarks placed by podiatrists were used by the designers as guidance to place digital landmarks in the orthotic design software. This approach facilitated the evaluation of consistency in landmark placement between experienced podiatrists. The scans, both with and without stickers were then sent to three different orthotic designers for creation of a total of eight orthotic designs each.

**FIGURE 6:**
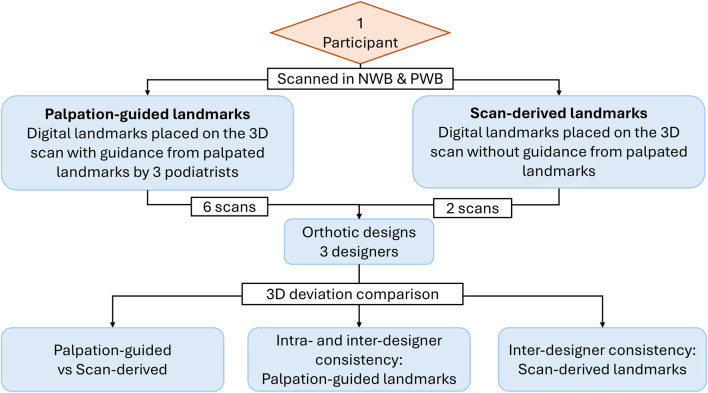
Flowchart of methodology to analyse consistency in orthotic designs (6 scans-landmarks placed by three podiatrists in two weight bearing positions, 2 scans-no landmarks placed in two weight-bearing positions).

To evaluate the consistency across different orthotic designs, the analysis was conducted under variable categories as listed in Figure 6.

- Influence of landmarking method on orthotic design consistency: In this category, orthotic designs based on palpation-guided landmarks were compared to those designed using scan-derived landmarks. This analysis aimed to determine if the landmarking method affected the consistency of orthotic designs. Since the orthotic designs were designed based on these landmarks, understanding how closely the resulting designs align within a defined tolerance (±1 mm) is critical for evaluating precision and repeatability of each approach. The weight-bearing condition was also considered to understand the impact of load on design consistency.
- Intra- and inter-designer consistency using palpation-guided landmarks: This part of the study aimed to estimate consistencies amongst podiatrists and designers and evaluated whether variability in landmark placement by different podiatrists influences the final orthotic design. The inter-designer consistency was analysed to understand whether different orthotic designers interpret the same landmarks similarly. The intra-designer consistency was analysed to understand whether a single designer produces consistent designs from different podiatrist’s palpated landmark on the same foot.
- Inter-designer consistency using scan-derived landmarks: This category assessed the orthotic designs based on scan-derived digital landmarks placed by designers as per their own discretion. This analysis helped to understand the design consistency across different designers when designed without manual palpation-guidance.

Additionally, the effect of weight-bearing was explored using a subset of orthotic designs with uniform landmarking (palpation-guided or scan-derived) to examine how foot deformation from NWB to PWB influenced the design consistency. This assessment provided insights into anatomical changes between NWB and PWB conditions and how designers interpret these changes in orthotic design.

#### Scanning process

**FIGURE 7:**
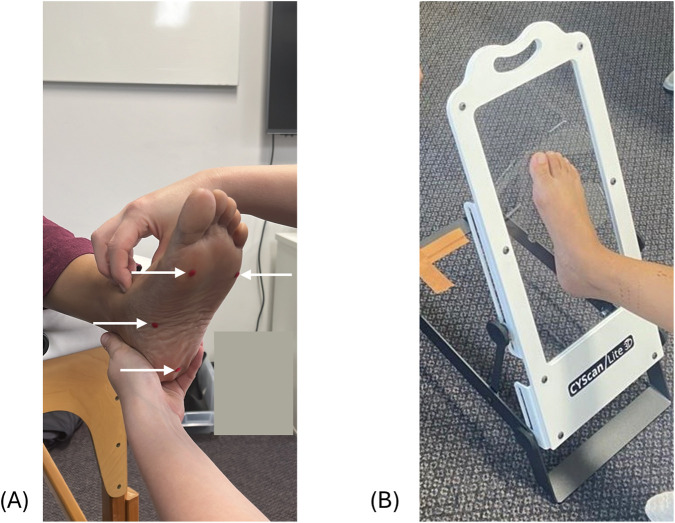
Scanning positions for orthotic consistency, **(A)** NWB and **(B)** PWB, (3D stickers on landmarks indicated with arrows).

For the second phase to assess consistency in orthotic designs, the foot was scanned using Truedepth phone application-based camera (Truedepth Camera; Apple, Inc., Cupertino, CA). The Truedepth phone application-based camera was chosen as this method is commonly used in clinical settings. The foot was marked with four landmarks using stickers, essential to design foot orthotics in the industrial orthotic insole design software. For the NWB position, the participant was asked to sit on a plinth with their feet extended beyond the edge with ankle placed in subtalar neutral position and for the PWB position, the participant was seated on a chair with the foot placed on a piece of Perspex glass angled at 45°. The scanning position is illustrated in Figure 7. Four different scans were obtained in each weight bearing condition. The first scan was performed without the placement of palpated landmarks, while the subsequent three scans involved the placement of anatomical landmarks by three different podiatrists after manual palpation for guidance.

#### Consistency analysis

To conduct a preliminary assessment of the consistency in orthotic design, the eight scans obtained in two different weight bearing conditions were sent to three professional orthotic designers. Six of the orthotics were designed based on guidance from palpated landmarks provided by the three podiatrists for each weight bearing condition. The remaining two scans were obtained without palpated landmarks to be used by the designers to digitally assign scan-derived landmarks as per their own discretion for designing orthotics. The orthotic designs were then compared using Geomagic Control X (version 2023.1.0, GeoMagic Inc., United States). Each digital orthotic design was loaded as an. STL file. The orthotic designs were then compared using the 3D compare module. The tolerance limit was set to ±1 mm denoting an acceptable value in the orthotic design industry (Redaelli et al., 2021). Arch height measurements were extracted from Rhino Grasshopper workflow to assess the actual difference in medial arch height. The 3D deviation analysis and arch height measurement were conducted to assess the consistency in orthotic designs.

### Statistical analysis

The statistical analysis was performed using SPSS Version 29.0 (SPSS inc., Chicago, IL, United States) and MS Excel. The error bars describe standard deviation.

The sample size calculation was based on a minimum acceptable 1CC reliability of 0.5, with an expected reliability of 0.8. The significance level was set at 0.05 (two-tailed) with a statistical power of 80%. As per the calculation, analysis of 18 unique foot scans are sufficient to achieve this power (Wnarifin, 2025; NCBI, 2025). An ICC value of 0.5 or higher indicates that the method is reliable. Specifically, values below 0.5 suggest poor reliability, values between 0.5 and 0.75 indicate moderate reliability, values between 0.75 and 0.9 reflect good reliability, and values above 0.9 represent excellent reliability at a confidence interval of 95%.

For the reliability of foot measurements, intra-Rhino-user reliability was assessed using a two-way, mixed effects, absolute agreement, single-rater Inter-Class Correlation (ICC) and inter-Rhino-user reliability was assessed using a two-way, random effects, absolute agreement, single-rater ICC in line with the McGraw and Wong classification (McGraw and Wong, 1996). All ICCs were reported with 95% confidence intervals to describe the precision of the reliability estimates.

## Results

This multi-user, multi-designer workflow has not previously been evaluated in orthotic research and enables direct comparison of measurement reliability and resulting design variability under controlled changes in landmarking method and weight-bearing condition.

### Reliability of foot measurements

**FIGURE 8:**
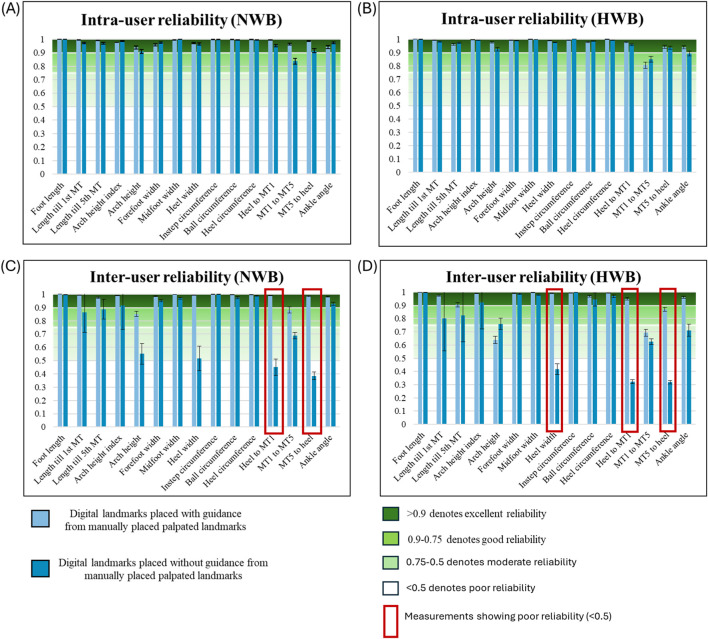
ICC representing intra- and inter-user reliability of foot measurements (N = 24). **(A)** Intra-user reliability (user A) in NWB condition, **(B)** Intra-user reliability (user A) in HWB condition, **(C)** Inter-user reliability (user A,B and C) in NWB condition, and **(D)** Inter-user reliability (user A,B and C) in HWB condition. Measurements demonstrating poor reliability are highlighted in red boxes. All ICCs were reported with 95% confidence intervals to describe the precision of the reliability estimates.

The ICC values are for intra- and inter-user reliability are graphically represented in Figure 8.

#### Intra-user reliability

With palpation-guided landmarks, ICC values demonstrated excellent intra-user reliability across all 15 foot measurements in both NWB and HWB conditions (Figures 8A,B) when using palpated landmarks (ICC = 0.939–1.000).

With scan-derived landmarks, NWB reliability (Figure 8A) remained generally high; however, a slight decline in ICC values was observed, particularly for the MT1 to MT5 distance. Despite this, these values still indicated good reliability. In HWB conditions (Figure 8B) with scan-derived landmarks, all measurements except for MT1 to MT5 and ankle angle maintained excellent reliability (ICC = 0.925–1.000), while the latter two showed good reliability.

#### Inter-user reliability

With palpation-guided landmarks, ICC values in the NWB condition (Figure 8C) ranged from 0.973 to 1.000 across all measurements, indicating excellent reliability for most measurements. Exceptions were noted for the arch point and MT1 to MT5 distance, which showed good reliability. In HWB conditions (Figure 8D), guided landmarking again resulted in generally high reliability, with excellent ICCs ranging from 0.905 to 1.000. The MT5 to heel distance demonstrated good reliability, while the arch point and MT1 to MT5 distance showed moderate reliability.

With scan-derived landmarks, the reliability between users became more variable. In the NWB condition (Figure 8C), measurements related to foot length, forefoot width, midfoot width, and circumferences retained good to excellent ICCs (0.916–0.999), while heel width and distances from the heel to MT1 and MT5 showed poor reliability. The arch point and MT1 to MT5 distances demonstrated moderate reliability. In HWB condition (Figure 8D), measurements such as arch height, foot length, and circumferences remained highly reliable. However, distances involving heel width and heel-to-MT points showed reduced ICC values, indicating poor reliability.

##### Pairwise inter-user reliability based on expertise

The inter-user ICC values also reflected the varying expertise of users:

- User A vs. User B: ICCs remained excellent (0.904–1.000) for most measurements. Moderate reliability was observed for the arch point and MT1 to MT5 distance, whereas heel width and heel point to MT5 showed good reliability.
- User B vs. User C: ICC values were excellent for standard foot dimensions and circumferences, but decreased for landmarks, such as the arch point and MT1 to MT5. Poor reliability was noted in heel width and heel to MT1 and MT5 distances.
- User C vs. User A: Similar trends were observed, with high ICCs for general foot shape but moderate to poor reliability for measurements directly related to landmarks.

### Consistency of orthotic designs

The consistency in the orthotic designs was analysed by performing 3D deviation analysis and comparing the deviation within tolerance values for the conformity of orthotic shapes. This phase of the preliminary study was designed to emulate the typical clinical and industrial orthotic design workflow in Australia, incorporating elements commonly used in practice. The smartphone-based scanner was selected due to its growing popularity, with partial weight-bearing (PWB) positioning reflecting the standard approach among podiatrists. The number and choice of anatomical landmarks were selected based on the specific requirements of the orthotic design software algorithm commonly used in the orthotic industry.

#### Influence of landmarking method on orthotic design consistency

**FIGURE 9:**
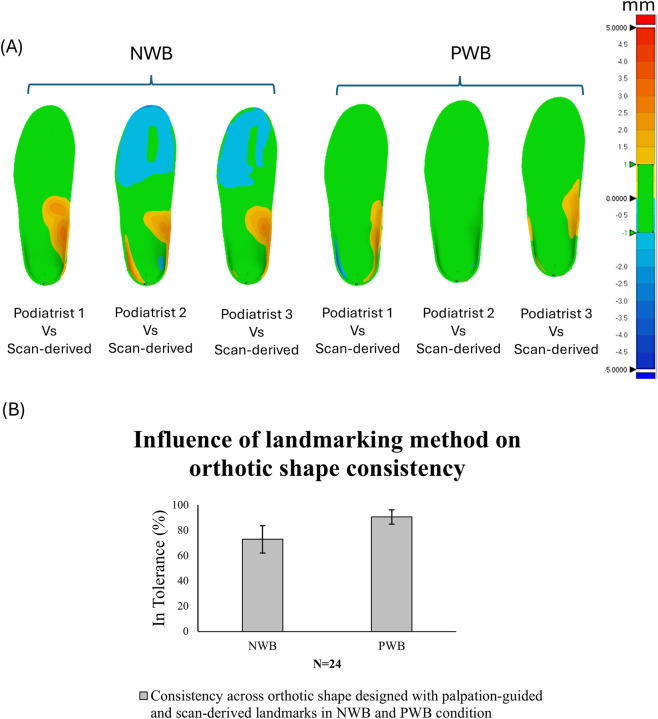
**(A)** 3D deviation analysis: Intra-designer consistency for palpation-guided vs. scan-derived landmarks. Green region in the orthotic designs indicates acceptable deviation, also referred to as “in tolerance” in which the orthotic designs are conforming within a range of ±1 mm. The red colour indicates all the positive values exceeding +1 mm and the blue region indicates all the negative values beyond -1 mm. **(B)** % similarity in orthotic designs obtained from palpation-guided vs. scan-derived landmarks.

To evaluate the consistency of orthotic designs for palpation-guided and scan-derived landmarks, the designs were compared by analysing the conformity of orthotic shape within a ±1 mm tolerance region. For the NWB position, on average 72.99% ± 10.85% of the orthotic designs fell within this tolerance region for all designers, increasing to 90.61% ± 5.65% in the PWB condition for all designers. The overall analysis for this comparison is illustrated in Figure 9 for both the weight bearing conditions.

##### Intra- and inter-designer consistency using palpation-guided landmarks

To determine whether podiatrist variability influenced design outcomes, palpation-guided landmarks placed by three different podiatrists were assessed. When different designers created orthotics using identical podiatrist landmarks, the inter-designer consistency was 59.21% ± 20.32% for NWB and 68.93% ± 14.65% for PWB. Conversely, when the same designer created orthotic designs using landmarks placed by different podiatrists (intra-designer variation), the consistency improved to 74.07% ± 11.16% for NWB and 85.03% ± 6.47% for PWB. The analysis for this comparison is illustrated in the Supplementary Figure S2 for both the weight bearing conditions.

##### Inter-designer consistency using scan-derived landmarks

For designs generated from scan-derived landmarks, the inter-designer consistency was 69.74% ± 14.33% in the NWB and 72.70% ± 13.64% in the PWB condition. The analysis for this comparison is illustrated in the Supplementary Figure S3 for both the weight bearing conditions.

**FIGURE 10:**
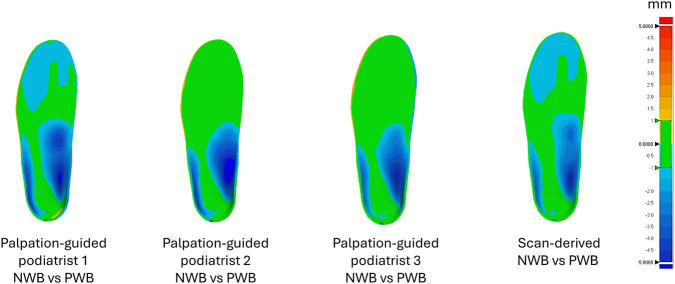
3D deviation analysis: Intra-designer consistency for NWB vs. PWB. Green region indicates acceptable deviation, also referred to as “in tolerance” in which the orthotic designs are conforming within a range of ±1 mm. The red colour indicates all the positive values exceeding +1 mm and the blue region indicates all the negative values beyond -1 mm.

Orthotic designs produced from the same landmark set by same designer under NWB and PWB conditions revealed regional height differences ranging from 2 mm to 8 mm, particularly in the arch and heel regions as illustrated in Figure 10. In terms of arch height landmark, the difference revealed that podiatrists palpated a mean positional change of 6.68 ± 0.84 mm from NWB to PWB, while designers estimated a smaller shift of 4.49 ± 1.85 mm based on scan-derived data.

## Discussion

Unlike previous studies that focused solely on scanner accuracy or foot measurement reliability in isolation, this study provides the first integrated assessment of how landmarking method, user expertise, and weight-bearing condition collectively influence foot measurements and orthotic design consistency. With advancement in technology, the orthotic industry is inclining towards switching the traditional tools and techniques with the digital ones for improved efficiency and scalability (Barrios-Muriel et al., 2020; Binedell et al., 2020; Kogler and Hovorka, 2021; Yuen et al., 2023; Senbekov et al., 2020). However, this transition has introduced newfound challenges, particularly in the standardisation of anatomical landmarking which forms the foundation for foot measurements and orthotic designs. With this shift, anatomical expertise resides with podiatrists while digital expertise lies with designers, creating a mismatch that challenges consistent landmarking and measurements. This study investigated the impact of palpation-guided versus scan-derived landmarking on the reliability of foot measurements (phase 1) and consistency of orthotic designs (phase 2). Across NWB and HWB conditions, intra-user reliability remained excellent for both methods, whereas inter-user reliability dropped substantially for scan-derived landmarks in clinically important measures such as arch height, MT1-to-heel, MT5-to-heel, and heel width. The findings from this study assist in clarifying whether design inconsistencies stem from user skill level, variability in landmarking methods, or phase-specific changes in foot morphology under different weight-bearing conditions. Notably, the observed low inter-user ICC values for plantar measurements highlight a critical limitation of relying on unguided digital landmark estimation in clinical and commercial orthotic workflows. The findings from this study are valuable for clinical and orthotic fabrication settings aiming to optimize their digital workflows, train staff effectively, and enhance outcome consistency across varying level of user expertise.

The reliability of foot measurements was assessed by three different users on 24 foot scans, scanned in NWB and HWB condition. The findings demonstrate that palpation-guided landmarks improve both the intra- and inter-user reliability when making digital foot measurements, especially in key measurements such as arch height, MT1 to hell, MT5 to heel and heel width. Intra-user reliability remained excellent for both approaches (>0.9), indicating that each user was consistent within their own repeated measurements. However, the inter-user ICC values dropped markedly for the scan-derived approach, particularly for MT1-heel, MT5-heel, and heel width, where the ICC decreased from 0.87-0.99 (with landmarks) to 0.31–0.55 (without landmarks). This marked reduction to poor inter-user agreement demonstrates that unguided, scan-derived landmark estimation (“digital guessing”) is inherently unreliable between operators, particularly for plantar measurements that lack clear visual reference points. Notably, arch height in NWB also showed reduced agreement (0.85 vs. 0.55), whereas in HWB, the ICC was slightly higher without landmarks (0.75 vs. 0.63), likely due to the more uniform foot contour under loading. These findings highlight that landmark visibility critically affects inter-user consistency for key clinical measures. In both weight bearing conditions, palpation-guided landmarks indicated higher ICC values, representing better reliability across same and different users. These results align with prior studies (Kouchi and Mochimaru, 2011; Aynechi et al., 2011; Dong et al., 2021; Gibelli et al., 2022; Weinberg et al., 2004), such as one conducted by Powers et al. (2022), which reported higher consistency for measurements generated from manual palpation. Similar trends have been observed in landmark placement across facial scans by Aynechi et al. (Gibelli et al., 2022), highlighting broader relevance of this issue.

For foot scans with scan-derived landmarks, where the user had to estimate the point in the digital scan, reliability was relatively low. The inclusion of user C, with a lower bound of anatomical expertise, highlights the dependency of scan-derived landmarking reliability on anatomical training rather than solely on software proficiency. Especially for users with limited anatomical knowledge, several key measurements such as arch point and inter-metatarsal distance demonstrated reduced reliability, emphasizing that accuracy of digital landmarks does not solely depend on the software but is also significantly influenced by the user’s clinical understanding. The lower reliability scores for the user with no anatomical training suggest that scan-derived landmarking may not be suitable for novice users lacking expertise, also reported by previous studies (Kouchi and Mochimaru, 2011; Kouchi et al., 1999). Regardless of 3D design experience, the translation of patient-specific anatomical data from clinical to industrial manufacturing settings is only effective when industrial orthotic designers are trained in anatomy and identification of bony landmarks. In addition, scan-derived landmarks may not fully substitute for anatomical palpation in certain pathologies, particularly in cases of foot swelling or in presence of lesions (Vega-et al., 2023; Rodriguez et al., 2023). This study was performed with healthy participants as a baseline. As such, the findings may not fully reflect the reduced landmarking reliability likely to occur in pathological feet (e.g., oedema or Charcot deformity), warranting further investigation in clinical populations In addition, the orthotic design consistency analysis was based on a single subject; therefore, the findings represent a preliminary assessment and should be interpreted with caution. Weight-bearing conditions affected measurement reliability differently across parameters. Flattening of the arch in HWB scans made the arch contour more uniform, slightly improving inter-user reliability compared to NWB, whereas NWB conditions showed higher variability, particularly in the plantar and hindfoot regions. These findings suggest that weight-bearing must be carefully considered when designing orthotics or assessing plantar geometry.

For a preliminary evaluation of the orthotic designs produced for palpation-guided and scan-derived landmarking methods, the consistency was assessed by measuring orthotic shape conformity within a tolerance region of ±1 mm (Redaelli et al., 2021). The consistency between orthotic designs based on palpated landmarks by podiatrists when compared to designs based on scan-derived landmarks demonstrated highest conformity for the PWB position compared to the NWB position. This marked increase in conformity under PWB conditions suggest that partial loading of the foot stabilises the soft tissue and the medial longitudinal arch,thereby reducing ambiguity in arch interpretation during the orthotic design process. These findings indicate that PWB scans may provide a more reproducible representation of foot geometry for digital orthotic design, particularly in the arch region where design discretion is greatest. The variation in NWB condition could be observed particularly in the arch height (up to 4 mm), which could be significant for the orthotic user, this effect was less visible (up to 2 mm) in the PWB condition. An error of up to 4 mm in arch height may substantially alter plantar load distribution, particularly in the MLA region where orthotic correction is most targeted. This error exceeds the acceptable tolerance value in the orthotic design industry (±1 mm) (Redaelli et al., 2021) and may contribute to discomfort, reduced compliance, or device rejection. These findings underscore the importance of both anatomical landmarking strategy and weight-bearing protocol when designing digital orthoses, as seemingly small geometric errors can translate into clinically meaningful outcomes.

In contrast, forefoot and hindfoot regions in the orthotic designs showed higher conformity regardless of weight-bearing condition. However, it is important to consider that the orthotic designs in the study were made by designers with several years of experience working with podiatrists. A similar trend was observed in inter-designer consistency, where providing all designers with identical palpation-guided landmarks (same podiatrist’s landmarks given to all designers) resulted in lower consistency than the intra-designer consistency for non-identical palpation-guided landmarks (landmarks placed by different podiatrists). These findings indicate that while differences in podiatrist landmark placement can introduce variability, experienced designers may reduce this impact through their ability to adapt to varied inputs. Additional findings from the consistency analysis are presented in detail in the Supplementary Material. It should be noted that the orthotic designs were not assessed through physical or experimental trials, representing a limitation of the study.

Both study phases indicate that anatomical knowledge plays a critical role in interpreting foot morphology. While palpation ensures reliable anatomical landmark identification, designing the corresponding arch height in the orthotic designs requires additional biomechanical expertise and a standardised design approach, especially in clinically critical region such as medial longitudinal arch. Further studies involving designers with varying experience levels (novices in training vs. experts) and multiple participants with varying foot shapes and pathologies is essential.

Orthotic designing has traditionally been a practical and experience-based approach, relying heavily on the expertise of Allied Health Professionals (Holtkamp et al., 2024). The digital era is reforming this process by enhancing reliability and consistency by reducing the variability associated with different users and their level of experience. This study shows that the digital design process still requires manual input from the designers and is still affected by human error. There is variation between orthotic designs created by different designers and there is variation in the placement of palpated landmarks by different podiatrists. While digital tools enhance workflow efficiency and scalability, tasks like landmark identification still require the clinical expertise of trained professionals (Wickramasinghe et al., 2022). If palpated landmarks are used, podiatrists and designers would benefit from more collaborative training to align their approaches and reduce variability. Also, emerging technologies such as ultrasound-based landmark detection, motion capture systems and AI-driven automated landmarking could enhance landmarking accuracy thus supporting more standardised orthotic design workflows (Rogati et al., 2021; Wu et al., 2018; Wang et al., 2018). However, their integration in the actual clinical practice currently remains limited due to the high cost, complexity and workflow compatibility, thus making them less viable in practical settings (Choo and Chang, 2023; Williams et al., 2016).

## Conclusion

This study provides the first integrated evaluation of how palpation-guided versus scan-derived landmarking, user expertise, and foot weight-bearing conditions collectively influence foot measurement reliability and orthotic design consistency. A common question while adopting digital technology for the purpose of orthotic designing is whether only to rely on manual palpation or entrust digital identification of anatomical landmarks in the 3D foot scans. This uncertainty leads to concerns about which approach ensures greater reliability and higher consistency. This study aimed to address this issue by analysing the reliability of foot measurement and consistency in orthotic designs with palpation-guided and scan-derived landmarks. The findings suggest that palpation-guided landmarks demonstrate enhanced reliability for general foot measurements and relative consistency of orthotic designs. However, the detailed consistency outcome showed mixed results due to variability introduced by the orthotic designer’s discretion. Variations in foot loading conditions influenced both measurement reliability and orthotic design consistency. Overall, palpation remains a valuable aid in this controlled study and is likely even more crucial in less controlled clinical settings. However, if palpation needs to be integrated with the digital designing methods, collaborative training between podiatrists and designers will ensure that clinical insight and technical expertise are effectively merged to improve the accuracy of arch height in orthotic designs.

## Data Availability

The original contributions presented in the study are included in the article/Supplementary Material, further inquiries can be directed to the corresponding author. The raw measurements, deviation analysis statistics, and statistical analysis used in the study will be made fully available to the public via QUT’s Institutional Research Data Finder (RDF) after the paper has been published. Through the RDF, non-institutional researchers may be granted access to these data after making a request to myself as the first author (Komal Chhikara, komal.chhikara@hdr.qut.edu.au) and owner of the RDF entries.
