# Supplementary material for: Is palpation essential in the digital era of orthotic designing?
Source: Front Bioeng Biotechnol. 2026 Feb 19;14:1648513. doi: 10.3389/fbioe.2026.1648513 (PMC12960551; doi:10.3389/fbioe.2026.1648513)
Supplement: Supplementary file 1 [file Supplementaryfile1.docx]

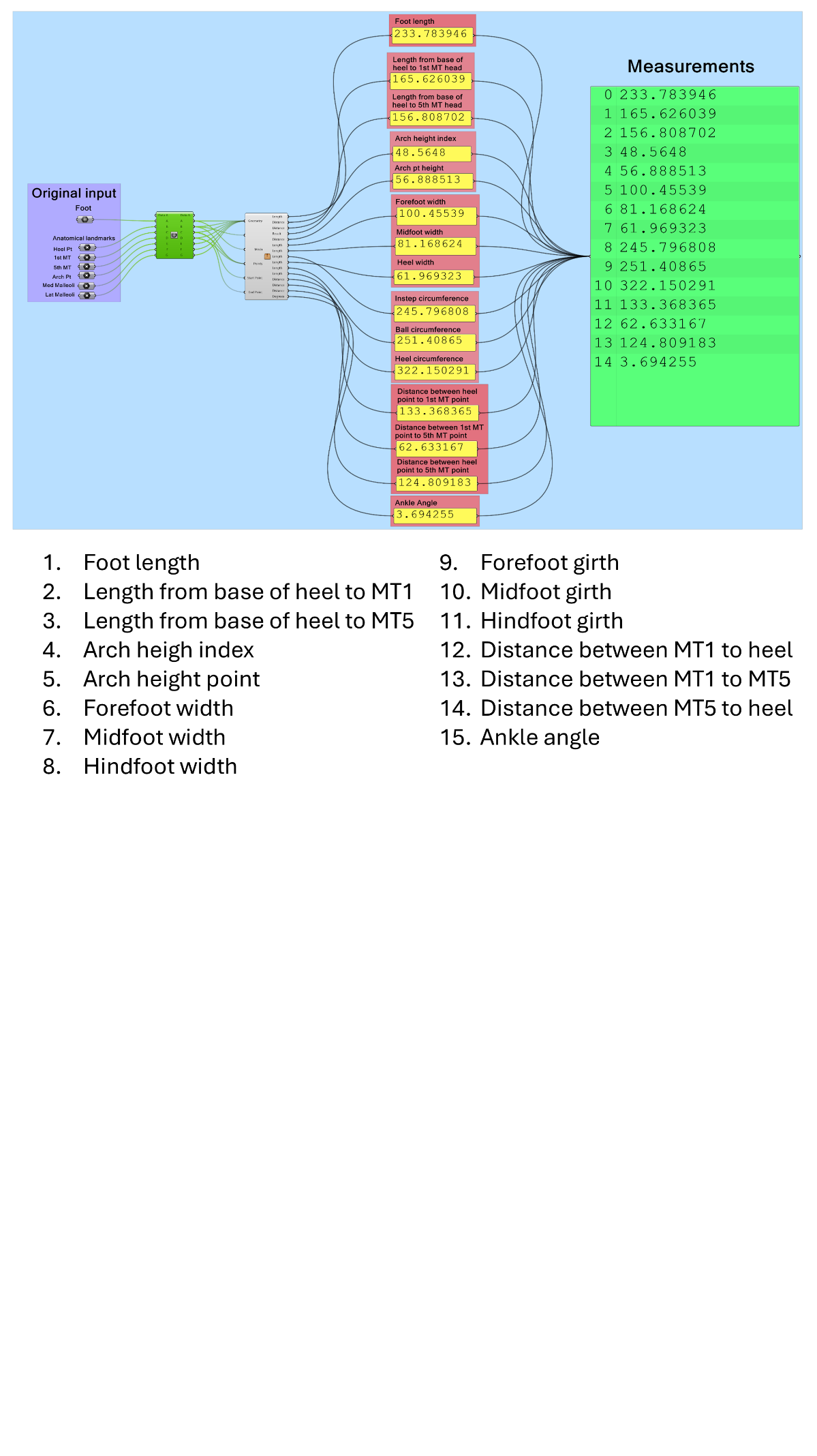


*Figure S1. Rhino-Grasshopper measurement workflow algorithm to calculate 15 measurements to evaluate user reliability*

**Result and discussion for orthotic shape consistency**


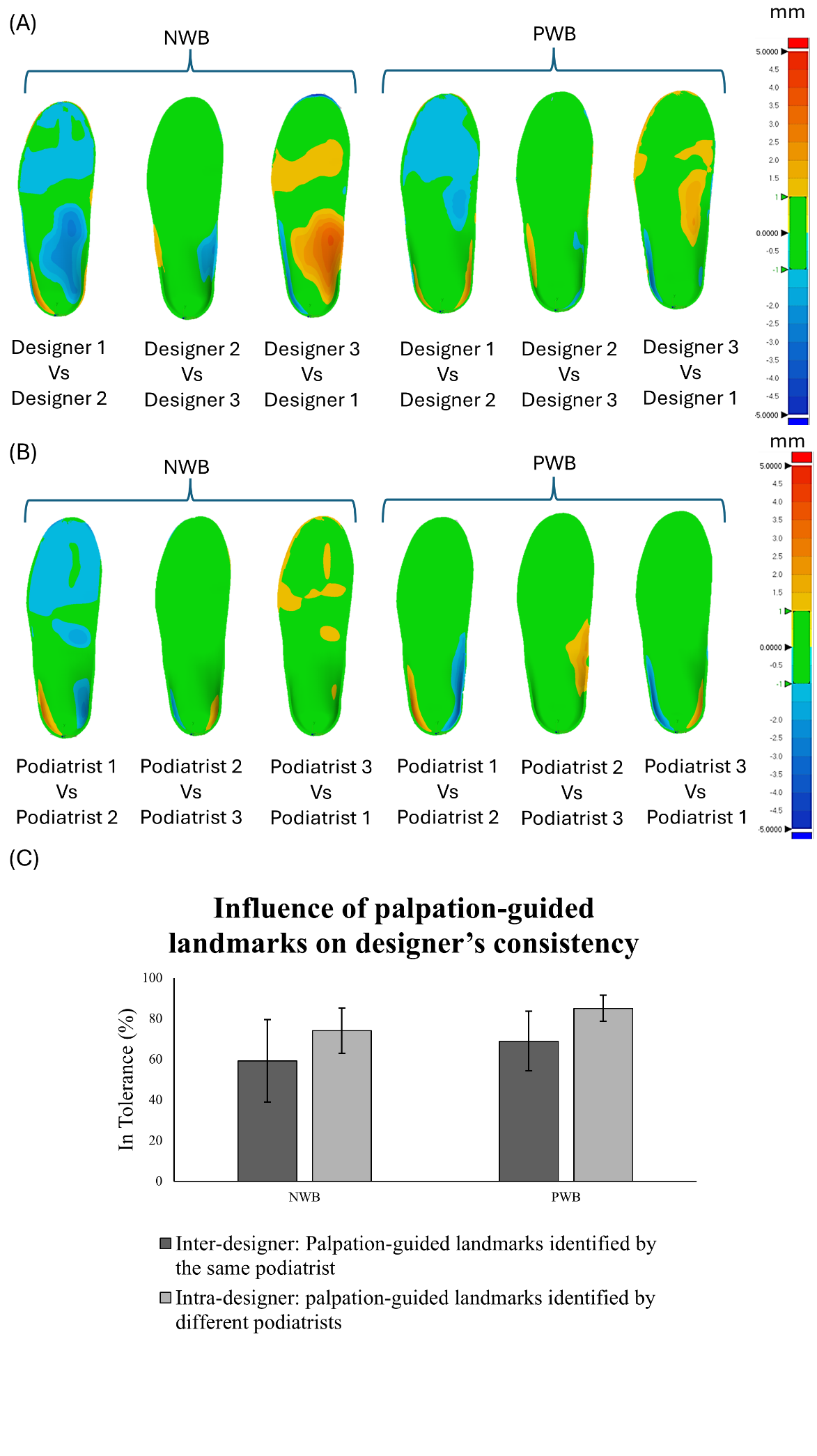


*Figure S2.* *(A) 3D deviation analysis: Inter-designer consistency for palpation-guided landmarks from same podiatrist. (B) 3D deviation analysis: Intra-designer consistency for palpation-guided landmarks from different podiatrists. Green region in the orthotic designs indicates acceptable deviation, also referred to as "in tolerance"​ in which the orthotic designs are conforming within a range of ±1mm. The red colour indicates all the positive values exceeding +1mm and the blue region indicates all the negative values beyond -1mm. (C) % similarity in orthotic designs obtained from identical and non-identical palpation-guided landmarks.*

Orthotic designs created by designers using their own discretion demonstrated scattered variation across all regions, not just arch (Figure S3). This accentuates the requirement of precision in placing landmarks as even small deviations could lead to inconsistencies in the orthotic designs. The variation in arch height could be observed up to 8mm between different weight bearing positions (NWB and PWB) in the foot. Also, the comparison of arch height reduction indicated that the designers may underestimate the medial arch collapse when relying on their own discretion without palpated landmarks.

These results suggest that while experience plays a role in landmark interpretation, the initial accuracy of input landmarks is critical for consistent orthotic output. While the study focused on consistency analysis using digital orthotic designs, additional investigation by analysing how the manufactured orthotics performed in the shoe is warranted for further validation.

*
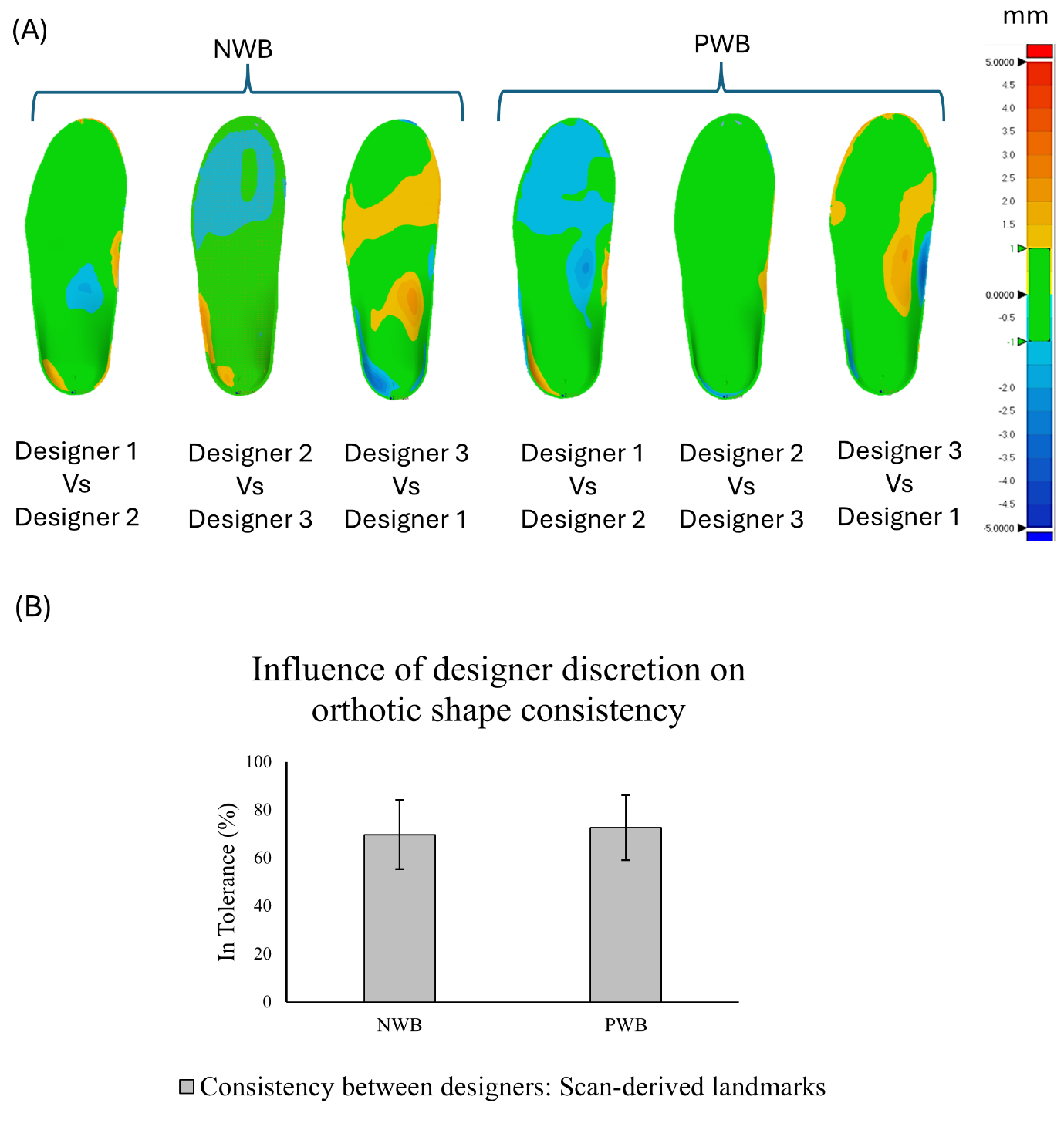
*

*Figure S3. (A) 3D deviation analysis: Inter-designer consistency for scan-derived landmarks. Green region in the orthotic designs indicates acceptable deviation, also referred to as "in tolerance"​ in which the orthotic designs are conforming within a range of ±1mm. The red colour indicates all the positive values exceeding +1mm and the blue region indicates all the negative values beyond -1mm. (B) % similarity in orthotic designs obtained from scan-derived landmarks*

This study demonstrated that the orthotic design consistency was generally higher in the PWB position suggesting more stability of foot morphology leading to less variations in weight bearing conditions. Overall, in all the comparisons, the regions showing highest variations were midfoot, particularly arch region, highlighting the crucial role of the arch landmark, which showed most variation amongst all four landmarks [56,57] . However, this effect was eliminated in the weight bearing condition, indicating that arch height could be better designed for load bearing foot scans. The comparison for consistency in orthotic designs for different weight bearing condition highlighted that scanning position plays a vital role for decision about arch height in the orthotics.
